# Research progress of dihydromyricetin in the treatment of diabetes mellitus

**DOI:** 10.3389/fendo.2023.1216907

**Published:** 2023-09-04

**Authors:** Ziyuan Wang, Zhuoran Cao, Zhiying Yue, Zhengfeng Yang

**Affiliations:** Precision Research Center for Refractory Diseases, Shanghai General Hospital, Shanghai Jiao Tong University School of Medicine, Shanghai, China

**Keywords:** dihydromyricetin, diabetes, diabetes complications, AMPK, research progress

## Abstract

Diabetic Mellitus (DM), a chronic metabolic disorder disease characterized by hyperglycemia, is mainly caused by the absolute or relative deficiency of insulin secretion or decreased insulin sensitivity in target tissue cells. Dihydromyricetin (DMY) is a flavonoid compound of dihydroflavonol that widely exists in Ampelopsis grossedentata. This review aims to summarize the research progress of DMY in the treatment of DM. A detailed summary of related signaling induced by DMY are discussed. Increasing evidence implicates that DMY display hypoglycemic effects in DM via improving glucose and lipid metabolism, attenuating inflammatory responses, and reducing oxidative stress, with the signal transduction pathways underlying the regulation of AMPK or mTOR/autophagy, and relevant downstream cascades, including PGC-1α/SIRT3, MEK/ERK, and PI3K/Akt signal pathways. Hence, the mechanisms underlying the therapeutic implications of DMY in DM are still obscure. In this review, following with a brief introduction of the absorption, metabolism, distribution, and excretion characteristics of DMY, we summarized the current pharmacological developments of DMY as well as possible molecular mechanisms in the treatment of DM, aiming to push the understanding about the protective role of DMY as well as its preclinical assessment of novel application.

## 1 Introduction

Diabetes Mellitus (DM) is a metabolic disease characterized by disordered lipid and carbohydrate metabolism. The prevalence of diabetes reported by the International Diabetes Federation shows that the number of adults aged 20-70 with diabetes worldwide is estimated to be 10.5%(536.6 million people)and is expected to rise to 12.2% (783.2 million) by 2045 (1, 2). DM patients will suffer pain from various DM complications, including kidney disease, diabetic foot, peripheral neuropathy, retinopathy and so on (3).

Diabetes, caused by a long-term combination of genetic and environmental factors, is mainly due to insulin deficiency or insufficient insulin secretion, leading to function disorders in glucose, lipid and protein metabolism (4). DM is largely classified into type I diabetes mellitus and type II diabetes mellitus (T2DM). T2DM has become the third most common non-communicable disease in the world. T2DM is characterized by insulin deficiency due to insulin secretion disorder and insulin resistance(IR) (5). The pathogenesis of T2DM may be caused by the massive generation of free radicals in response to high blood glucose and high free fatty acids, which leads to oxidative stress and inflammatory responses. The long-term inflammatory responses and the activation of oxidative stress-associated signaling pathways contribute to IR and impaired insulin secretion, thereby leading to the clinical symptoms of DM (6, 7). Until now, the etiology and pathogenesis for DM have not been fully clarified.

**Figure 1:**
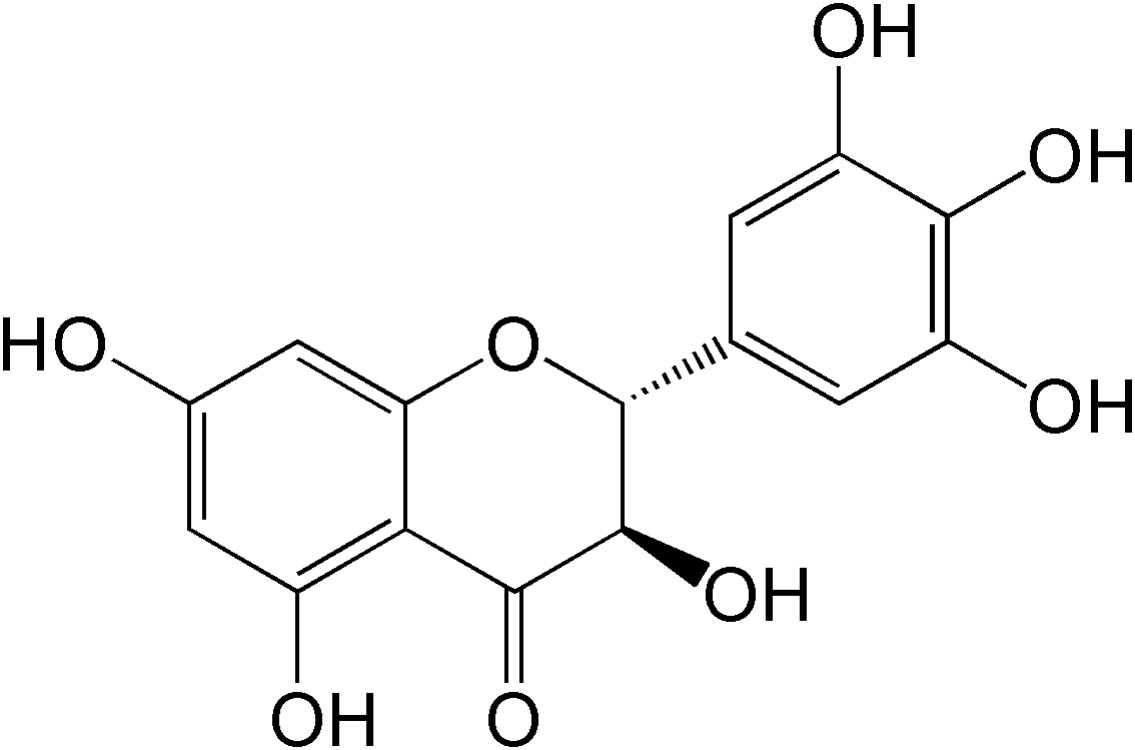
The chemical structure of DMY.

Dihydromyricetin (DMY), a 2,3-dihydroflavonol compound (**Figure 1**), is the main bioactive component isolated from the tender stem and leaves of the Chinese medicinal plant *Ampelopsis grossedentata* (*A. grossedentata*), which exert various biological effects, including anti-alcohol intoxication, anti-inflammatory, antibacterial, antioxidant, anti-tumor properties, as well as lipid and blood glucose-regulating effects (8, 9).

Pharmacokinetic studies have revealed that the half-life of DMY is approximately 3.70 ± 0.99 hours and the total time DMY remained in the body is around 5.98 ± 0.58 hours when DMY is orally administered at a dose of 20 mg/kg (10). Furthermore, once DMY is absorbed into the bloodstream, it would be easily metabolized and decomposed through methylation, sulfation, or reduction reaction. Fan et al. reported that after oral administration, unmetabolized DMY quickly distributes throughout various tissues, especially in the gastrointestinal tract. Based on *in vitro* metabolism studies, they also discovered that most of the original compounds and metabolites are excreted through feces, with only a small amount of original compounds being absorbed through the small intestine. These absorbed compounds enter the bloodstream through the portal vein, and reach the liver. In the liver, they undergo further processing through glucuronidation, sulfation, and methylation, resulting in phase II metabolites that are either absorbed by the body or excreted via urine (11). Due to extensive biotransformation occurring in the gastrointestinal tract and bloodstream, the oral bioavailability of DMY is relatively low (12). In short, they detected eight metabolites and proposed five metabolic pathways, including reduction, dehydroxylation, methylation, glucuronidation and sulfation.

**Figure 2:**
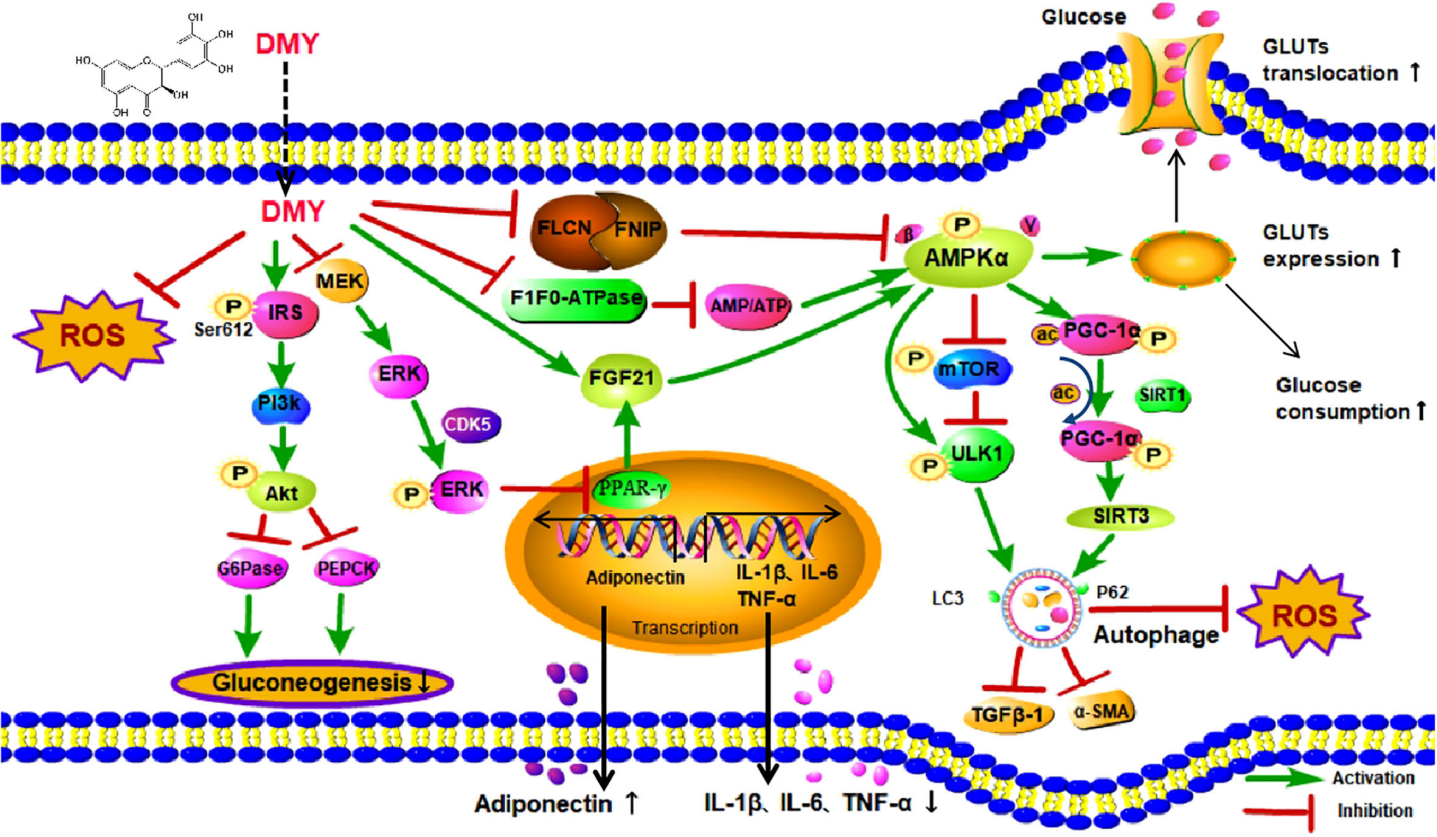
The effects and mechanisms on diabetes mellitus. DMY, Dihydromyricetin; ROS, Reactive oxygen species; IRS,insulin receptor substrate; MEK, mitogen-activated protein kinase kinase; ERK, extracellular regulated protein kinases; PI3K, phosphatidylinositol 3 kinase; Akt, protein kinase B; G6Pase, glucose-6-phosphatase; PEPCK, phosphoenolpyruvate carboxykina se;CDK5, cyclin dependent kinase-5; FLCN, Folliculin; FNIP1,Folliculin Interacting Protein 1; AMP, Cyclic Adenosine monophosphate; ATP,adenosine-triphosphate; FGF21, Fibroblast Growth Factor-21; PPARγ, peroxisome proliferator-activated receptor γ; IL-1β, Interleukin-1β; IL-6, Interleukin-6; TNF-α, tumor necrosis factor-α; AMPKα, Adenosine 5′-monophosphate activated protein α; mTOR, mammalian target of rapamycin; ULK1,unc-51-like kinase 1; TGFβ-1, transforming growth factor-β 1; α-SMA,α-Smooth muscle actin;PGC-1α, Peroxisome proliferator activated receptor co-activator-1α; SIRT1, Sirtuin1; SIRT3, Sirtuin3; P62, sequestosome 1; Gluts, Glucose transporters.

Recently, both clinical and experimental studies have illustrated that DMY possesses preventive and therapeutic effects on DM and its complications (13). Mechanistically, DMY has been reported to be involved in improving glucose and lipid metabolism, alleviating oxidative stress as well as attenuating inflammatory response, however, the exact molecular mechanism of DMY in DM is still not fully elucidated. Previous studies have shown that DMY was able to activate AMP-activated protein kinase (AMPK) signal pathway to perform a potential anti-diabetic effect, with mechanism related to AMPK/mTOR/PGC-1α and MEK/ERK signaling pathways. The review aims to summarize the role and molecular mechanisms of DMY in the treatment of DM and its complication and all the effects are listed in **Figure 2** and simplified in **Table 1**.

**Table 1:** Studies on the effects of DMY on DM.

Disease	Drugs	Cell lines/Animal models	Concentration/time	Results	Effect	References
Diabetic Cardiomyopathy	DMY	Streptozotocin induced Diabetic Mice	Intragastric gavage with 100 mg/kg/d,14 weeks	Becilin-1,LC3-II,LC3-II/LC3-I,Atg7↑,AMPK,ULK1↑, p62↓	Induce autophagy	14
Diabetic Nephropathy	DMY	Streptozotocin induced Diabetic Nephropathy Mice	Intragastric gavage with 50,100 mg/kg/d,8 weeks	AMPK,p-AMPK/AMPK,LC3 I/LC3II,Beclin-1↑; TGF-β1,α-SMA, mTOR↓	Induce autophagy	15
Diabetic angiopathy	≥98% DMY	HUVECs	0.1, 0.5, and 1 μM for 2 h.	LC3-II,Beclin1,Atg5↑; AMPK↑;,mTOR↓,p62↓	Induce autophagy,alleviate the endothelial cells	16
		PA-treated L6 myotubes	0.1, 0.5, and 1 μM for 2 h.	LC3-II,Beclin1,Atg5↑	Induce autophag, improve SMIR	17
		HFD-fed rats	Intragastric gavage with 10,50,100 mg/kg/d,8 weeks	Sequestosome 1↓,autophagosome↑,AMPK↑,TSC2↑;ULK1↑,mTOR↓,P70S6K↓,AMP/ATP↑		
T2DM	≥98% DMY	PA-treated C2C12 myotubes	0.1, 0.5, and 1 μM for 2 h.	P-AMPK↑,p-PGC-1α↑,p-Sirt3↑,LC3-II↑,P62↓	Induce autophagy, improve SMIR	17
		Sirt3-/- mice	Intragastric gavage with 50 mg/kg/d,3 months			
T2DM	DMY	HFD-fed rats	Oral 100,200,400 mg/kg/d,8 weeks	AMPK↑; GLUT 1↑;PEPCK↓, IRS-1↓; Akt↑; GSK-3β↓,G6Pase↓	Enhance insulin sensitivity and glucose uptake; inhibit glucose production	18
obesity-induced slow-twitch-fiber	≥98% DMY	HFD-fed and ob/ob mice	100 mg/kg/d,16 weeks	FLCN↓, FNIP1↓,AMPK↑; PGC-1α↑, Myh7↑;p-Akt, p-IRS-1↑	Attenuate the reduction of slow-twitch-fiber; ameliorate insulin resistance	19
		PA-treated C2C12 myotubes	0-5 μM for 16 h.			
T2DM	≥98% DMY	PA-treated C2C12 myotubes	1,5 or 10 μM for 24 h.	PPARγ↑, FGF21↑,AMPK↑	Enhance insulin resistance	20
			10 µM for 6, 12 or 24h			
T2DM	≥95% DMY	db/db Mice	Oral 0.5 g and 1.0 g/kg,8 weeks	Fasting blood glucose,serum insulin, HOMAIR↓;p-IRS-1^Tyr612^↑	Improve Insulin Sensitivity	21
T2DM	≥98% DMY	Zucker diabetic fatty rats	Intragastric gavage with 50,100 or 200 mg/kg/d,8 weeks	Reduce fasting blood glucose; p-PPARγ273↓,CDK5,p-ERK1/2↓;TG↓,HDL-C↑	Improve insulin sensitivity, ameliorateinsulin resistance	22
T2DM	DMY	Embryonic fibroblast 3T3-L1 cells	1, 3, 10 µM for 0.5,1,2 h.	Adiponectin↑; Insulin sensitivity↑;p-PPARγ273,p-ERK ↓	Dose dependently enhance glucose uptake and decrease adipogenesis in adipocytes	23
T2DM	DMY	Streptozotocin induced Diabetic SIRT3^-/^-mice	Intragastric gavage with 250 mg/kg/d, 12 weeks	Fasting blood glucose,HbA1c↓,ROS↓;SIRT3,SOD2,mtDNA copy number↑	Inhibit ROS	24
T2DM	≥95% DMY	Tetraoxypyrimidine induced Diabetic SIRT3^-/^-mice	Intragastric gavage with 50,100,150 mg/kg/d,4 weeks	SOD,GSH-Px↑;MDA,NOS↓	Enhance the antioxidant capacity,alleviate the damage of liver and pancreatic β cells	25
T2DM	≥98% DMY	Streptozotocin induced Diabetic Mice	Intragastric gavage with 125, 250 mg/kg/d, 16 weeks	BDNF↑, Cognitive function↑;TC,TG,LDL↓,HDL↑;Oxidative stress in the hippocampus of T2DM mice↓	Suppress oxidative stress	26
Diabetic nephropathy	DMY	Streptozotocin induced Diabetic Nephropathy Mice	Intragastric gavage with 100 mg/kg/d, 10 weeks	Fasting blood glucose↓;PI3K/Akt/mTOR↓,PTEN↑,Col IV, α-SMA, p62↓;LC3-II/I,Beclin 1↑	Induce autophagy	27
		NRK-52E cells	1 μM for 24 h or 48 h			
Diabetic retinopathy	DMY	ARPE-19 cells	30, 100 and 300 μM for 3 h prior to high glucose treatment	miR-34a↓,ROS↓;SOD,CAT,GSH↑	oxidative stress and Alleviate apoptosis	28
Diabetic neuropathic pain and Depression	≥98% DMY	Streptozotocin induced Diabetic Mice	Injected intraperitoneally with 30 mg/kg/d, 2 weeks	BDNF/Trk in the hippocampus↑;BDNF/Trk in the spinal cord and DRG↓; IL-1β,TNF-α in the hippocampus, spinal cord and DRG↓;Cognitive function↑,	Alleviate diabetic cognitive dysfunction	29
Diabetic neuropathic pain and Depression	≥98% DMY	Streptozotocin induced Diabetic Mice	Injected intraperitoneally with 30 mg/kg/d, 2 weeks	P2X7 receptor↓,ERK1/2↓;IL-1β,TNF-α↓	Alleviate Diabetic Neuropathic Pain and Depression comorbidity symptoms	30
Diabetic cardiomyopathy	DMY	Streptozotocin induced Diabetic Mice	Intragastric gavage with 100 mg/kg/d, 14 weeks	MDA↓,SOD, GSH-Px↑;IL-6, TNF-α↓;ATP content, CS activity, complex I/II/III/IV/V activities↑;LVEF, LVFS, ± LV dp/dt↑,cleavedcaspase-3, cleaved caspase-9↓;Bcl-2↑, Bax↓, Bax/Bcl-2 ratio↓;LC3 II/LC3 I,Beclin1,Atg↑,P62↓,p-AMPK, p-ULK1↑	Attenuate oxidative stress, reduce the levels of inflammation factors, improve mitochondrial function, inhibit cardiac apoptosis, and restore autophagy	14
Diabetic cardiac insufficiency	≥98% DMY	Streptozotocin induced Diabetic Mice	Intragastric gavage with 250 mg/kg/d, 8 weeks	HMGB1↓,p-NF-κB p65↓;EF, FS, SV↑; LVIDd, LVIDs↓;TG↓,HDL-C↑	Reduces the inflammation	31
T2DM	DMY	HFD-fed mice	Intragastric gavage with 200 mg/kg/d, 12 weeks	IL-1β, IL-6, TNFα, MCP1↓; levels of glucose, insulin, total triglycerides, total cholesterol, free fatty acids↓	Enhance insulin sensitivity	32
		3T3-L1 cell	1 μM for 5 days	NF-κB, JNK↓;p-AMPK, p-IRS-1, p-AKT, GLUT4↑;Ca^2+^↑,CaMKK↑		

↑ stands for up-regulation; ↓ stands for down-regulation.

## 2 Molecular mechanism of DMY in the treatment of DM

### 2.1 DMY treats DM by activating the AMPK signaling pathway

#### 2.1.1 DMY activates AMPK-mTOR- autophagy

AMPK, a key energy stress sensor, is activated in response to an increased AMP/ATP ratio during various stress conditions, such as hypoxia, hypertrophy, hypoglycemia, low blood glucose levels, and other stressors (33). This crucial signaling pathway plays a role in distinguishing the distinct or similar regulation of cellular responses across different tissues and cell types.

Recent studies have revealed AMPK’s positive regulation of autophagy, which involves inhibiting the activity of the mammalian target of rapamycin (mTOR) complex and activating phosphorylated unc-51-like kinase 1 (ULK1) (34). Autophagy is essential for maintaining heart structure, function, and cardiomyocyte integrity. Additionally, AMPK has been linked to autophagy in other contexts, such as diabetic cardiomyopathy, where it may protect cardiomyocytes in diabetes through Bcl-2 dissociation from Beclin1 when activated (35). The alteration of AMPK activity in diabetic mice influences cardiac autophagy, as indicated by changes in the LC3II/LC3I ratio, Beclin1 expression, and autophagy-related gene 7 (Atg7) levels, all of which were reversed by DMY treatment (14).

In the context of diabetic complications, DMY has shown potential in various tissues. In renal tissues of diabetic rats, DMY increased AMPK protein expression levels, leading to improved glomerular extracellular matrix deposition and renal fibrosis, possibly through promoting autophagy and inhibiting the expression of transforming growth factor-beta (TGFβ-1) and Alpha-smooth muscle actin (α-SMA) (15). In human umbilical vascular endothelial cells (HUVEC) exposed to high glucose-induced injury, DMY up-regulated autophagy through the AMPK/mTOR pathway (16). Additionally, in skeletal muscle cells, DMY enhanced insulin sensitivity by activating the AMPK/mTOR/ULK1 pathway, which induced autophagy (36). Overall, the AMPK/mTOR signaling pathway offers promising therapeutic avenues for diabetes treatment by targeting autophagy processes. Nevertheless, further research is needed to fully elucidate the distinct and common mechanisms through which AMPK regulates autophagy across different tissues and cell types.

#### 2.1.2 DMY activates AMPK-PGC-1α-SIRT3-autophagy

AMPK is a crucial intracellular serine/threonine-protein kinase, functioning as an energy stress sensor. It becomes activated by an increased AMP/ATP ratio during various stress conditions, such as hypoxia, hypertrophy, hypoglycemia, low blood glucose levels, and other stressors (33). Once activated, AMPK phosphorylates CREB (cAMP response element-binding protein), leading to the activation of peroxisome proliferator-activated receptor (PPARγ) coactivator-1α (PGC-1α) and downstream target Sirtuin 3 (SIRT3). PGC-1α serves as a transcriptional co-activator closely associated with the body’s energy metabolism, while SIRT3 induces autophagy through mitochondrial protein deacetylation, specifically mitochondrial autophagy, playing critical roles in cellular functions (37–39).

In a study conducted by Shi et al., DMY (a specific compound) was found to increase AMPK phosphorylation in skeletal muscle, subsequently upregulating PGC-1α expression and activating SIRT3. To validate the pathway’s role, the researchers used PGC-1α or AMPK siRNA transfection, resulting in reduced DMY-induced SIRT3 expression and suppressed autophagy in C2C12 myotubes. Consistent results were observed in skeletal muscle tissues of DMY-fed Stirt3^-/-^ mice, confirming the involvement of the AMPK-PGC-1α-SIRT3 pathway (17).

These findings imply that DMY promotes autophagy, partially via activating the AMPK-PGC-1α-SIRT3 signaling pathway, thereby enhancing skeletal muscle insulin sensitivity in both *in vitro* and *in vivo*.

In conclusion, this study highlights the significance of the AMPK/PGC-1α/SIRT3 signaling pathway as a potential multi-targeted therapeutic approach for diabetes mellitus (DM) treatment. Furthermore, it provides valuable insights into the mechanisms underlying the anti-diabetic effects of DMY, emphasizing the essential role of AMPK-related signaling pathways in promoting autophagy in skeletal muscle cells.

#### 2.1.3 DMY promotes translocation of GLUT1 from the membrane by activating AMPK signaling

Glucose transporter (GLUTs) is the major carrier of glucose transport in mammalian cells. AMPK serves as a crucial energy sensor to regulate lipid, cholesterol and glucose metabolism, from which AMPK activation leads to an increase in lipid and glucose catabolism (40). While a decrease in glucose production has advantageous effects on glucose homeostasis and peripheral insulin sensitivity (41). The overexpression of constitutively active AMPK in skeletal muscle cells leads to increased glucose uptake, accompanied by the trans-localization of GLUT1 and GLUT4 to the plasma membrane. This enhancement in glucose uptake and utilization further benefits skeletal muscle function. (42). Previous reports have confirmed the translocation of GLUT2 to the plasma membrane via the AMPK pathway in the liver (43). There have been studies showing that the activation of AMPK is correlated with the enhancement of glucose transport mediated by plasma membrane localized Glut1 activation (44). In another study, it was shown that DMY improves insulin resistance in rats fed with a high-fat diet (HFD) by facilitating the translocation of GLUT1 from the cytosol to the membrane. This effect is achieved through the activation of AMPK at Ser172 and AS160 signaling, which leads to an increase in glucose uptake (18).

### 2.2 DMY improves PI3K/Akt signal by activating AMPK

Insulin primarily exerts its action through the insulin receptor substrate (IRS)-inositol phosphate kinase (PI3K)-protein kinase B (Akt) pathway (45). Liang Le et al. discovered that DMY downregulates the expression of G6Pase and PEPCK through the IRS/PI3K/Akt pathway, inhibiting gluconeogenesis and reducing glucose production (18, 46).

In studies involving fatty db/db mice, oral administration of DMY reduced fasting blood glucose, serum insulin levels, and insulin resistance index levels. The mechanism behind this effect appears to involve DMY inducing intracellular transduction of insulin signaling by up-regulating IRS-1 (Y612) tyrosine phosphorylation, mitigating insulin resistance and pancreatic fibrosis in the db/db mice (21).

Furthermore, various investigations have consistently indicated a direct correlation between insulin-stimulated glucose disposal rate and a decrease in the proportion of slow-twitch (type I) fibers, leading to reduced oxidative metabolism in skeletal muscles (46).

Folliculin (FLCN), a protein associated with kidney cancer, has been recently linked to AMPK regulation through the FLCN/Folliculin Interacting Protein 1 (FNIP1) complex and its interacting protein FNIP1. This complex acts as a negative regulator of AMPK activity, impacting the expression of PGC-1α (47).

In a study by Zhou et al., palmitic acid-induced C2C12 muscular tube exhibited a decrease in the expression of slow-twitch fiber-specific Myh7, along with increased levels of FLCN and FNIP1, AMPK inactivation, and reduced PGC-1α expression. However, DMY treatment reversed this phenomenon, significantly reducing palmitic acid-induced insulin resistance. This effect was evident by increased levels of p-Akt and p-IRS-1, as well as enhanced glucose uptake (19). These findings suggest that DMY may counteract the obesity-induced decrease of slow-twitch fibers by regulating the FLCN-FNIP1-AMPK signaling pathway, ultimately improving skeletal muscle insulin resistance in obese patients.

PPARγ, a member of the type II nuclear hormone receptor superfamily, plays a key role in the regulation of glucose and lipid metabolism. PPARγ is considered a clinically proven therapeutic target for T2DM (48). PPARγ agonists induce the expression of Fibroblast Growth Factor 21 (FGF21), further enhancing PPARγ activity in adipocytes (49). Additionally, FGF21 activates AMPK and enhances mitochondrial function and oxidative capacity, ultimately improving glucose tolerance (50). Zhou et al. reported that DMY activates PPARγ, leading to increased glucose uptake capacity in palmitic acid-induced insulin-resistant L6 myotube cells in a dose-dependent and time-dependent manner. Furthermore, DMY markedly up-regulated the expressions of p-IRS-1, p-Akt, p-AMPK, and FGF21 (20). These findings suggest that DMY may act as a possible agonist of PPARγ and enhance insulin sensitization by activating PPARγ and subsequently regulating the FGF21-AMPK signaling pathway.

### 2.3 DMY promotes adiponectin secretion through MEK/ERK signaling pathway

Adiponectin is a crucial hormone predominantly synthesized and secreted by mature adipocytes, known for its antidiabetic, antiatherogenic, and anti-inflammatory functions. It exerts its effects by binding to adiponectin receptors (adipoR1 and adipoR2), leading to increased fatty acid oxidation, reduced hepatic glucose production, and improved insulin sensitivity (51). However, in the context of obesity, adipocytes become hypertrophic, which results in reduced secretion of adiponectin into the plasma and an imbalance in glucose homeostasis.

Moreover, the increased expression of adipose inflammatory cytokines leads to the impairment of insulin signaling pathways and reduced insulin-dependent glucose uptake, which further highlights the crucial role of adiponectin in insulin resistance (IR), type 2 diabetes mellitus (T2DM), and metabolic syndrome (52).

In their study, Lei et al. explored the effects of DMY on glucose uptake and adiponectin secretion in adipocytes. They observed that DMY treatment enhanced glucose uptake capacity in Zucker diabetic fatty rats and stimulated adipocytes to secrete adiponectin FGF21. This secreted adiponectin FGF21 demonstrated anti-diabetic efficacy by inhibiting extracellular signal-regulated kinase 1/2 (ERK)/cyclin-dependent kinase-5 (CDK5)-mediated PPARγ phosphorylation at Ser273 in adipose tissues (22). Consequently, DMY effectively delayed the onset of hyperglycemia and improved obesity-associated insulin resistance in diabetic rats without inducing weight gain.

Moreover, Liu et al. investigated whether DMY mitigates insulin resistance by inhibiting PPARγ phosphorylation at Ser273 via the MEK/ERK pathway in mouse fibroblast cells using the 3T3-L1 cell line. Their results showed that dexamethasone-treated adipocytes supplemented with DMY exhibited reduced insulin sensitivity for glucose uptake and decreased adipogenesis in a dose and time-dependent manner. Notably, pre-treatment of the PPARγ inhibitor, GW9662, effectively blocked the improvement of insulin resistance by DMY in differentiated adipocytes. Furthermore, both DMY and the mitogen-activated protein kinase (MEK) inhibitor, PD98059, significantly enhanced glucose uptake and adiponectin secretion in adipocytes (23). These findings suggest that DMY enhances glucose uptake in adipocytes by inhibiting the MEK/ERK pathway, which consequently down-regulates PPARγ phosphorylation at Ser273.

In summary, these studies demonstrate that DMY may have potent effects in reducing blood glucose levels, improving insulin sensitivity, and ameliorating lipid profiles. Agents that promote adiponectin secretion, such as DMY, could potentially be valuable candidates for treating metabolic diseases, including T2DM.

### 2.4 DMY reduces ROS production

Oxidative stress refers to the critical imbalance between intracellular reactive oxygen species (ROS) production and scavenging in the body (53). For diabetic patients, oxidative stress and lipid peroxidation, caused by elevated levels of oxygen free radicals, play a significant role. In islet β cells, antioxidant enzymes function to reduce oxidative damage directly by decreasing the production of ROS and free radicals, protecting the cells from harm (54, 55). In a recent report, alloxan and/or STZ, extensively used in establishing T2DM animal models, mainly increase the production of ROS and inhibit free radical defense system, making ROS directly injure islet β cells, finally resulting in necrosis of pancreatic β cells, thus leading to hyperglycemia and inducing the occurrence of diabetes (56). In a study by Hua and his colleagues, male C57BL/6 mice, wild-type mice and SIRT3^-/-^ mice were injected with STZ for 5 consecutive days (60 mg/kg/day. After a fortnight, DMY was given 250 mg/kg by gavage once a day for 12 weeks. They found that DMY treatment decreased fasting blood glucose and glycosylated hemoglobin level, improved endothelium-dependent relaxation of diabetic mice thoracic aorta, inhibited oxidative activity and ROS production, and enhanced SIRT3 and superoxide dismutase 2 protein expression (24). The results suggested that DMY improved profound endothelial dysfunction in diabetic mice via inhibiting oxidative stress and the production of ROS in a SIRT3-dependent manner.

Furthermore, high activities of superoxide dismutase (SOD), glutathione peroxidase (GSH-Px), and catalase (CAT) contribute significantly to reducing the accumulation of intracellular ROS (57). Recently, Xiao et al. reported that DMY markedly increased the activities of antioxidative enzymes SOD and GSH-Px in the serum, liver, and pancreas of diabetic mice. Additionally, DMY decreased the levels of MDA and nitric oxide synthase, suggesting that it enhanced the antioxidant capacity of diabetic mice. This effect led to alleviation of liver and pancreatic β cell damage and promotion of insulin synthesis (25).

Moreover, Ling and colleagues demonstrated that DMY significantly decreased the production of MDA, increased the anti-oxidant defense system, including SOD, GSH-Px, and CAT, and inhibited the production of ROS in the hippocampus of T2DM mice. These effects resulted in reduced glucose and lipid metabolism, improved mitochondrial function, and ultimately ameliorated cognitive impairment (26). Guo et al. measured blood glucose, serum insulin and renal superoxide compound levels after DMY treatment in each mouse. They found that DMY significantly reduced MDA content and increased the activity of SOD in the kidney, DMY promoted autophagy and improved renal interstitial fibrosis in diabetic nephropathy (DN) by regulating the miR-155-5p/phosphatase and tensin homolog deleted on chromosome ten (PTEN) signaling pathway and phosphatidylinositol 3-kinase (PI3K)/protein kinase B (AKT)/mammalian target of rapamycin (mTOR) signaling pathway, suggesting that DMY had a protective effect in early kidney injury of diabetic nephropathy, which may be associated with improvement of anti-oxidative capability in the kidney (27). Recent studies have found that the ROS content in high-glucose stimulated ARPE-19 cells is significantly decreased, while the activities of SOD and CAT antioxidant enzymes, the concentration of GSH are both enhanced. Hence DMY has a protective effect on HG-induced oxidative stress of ARPE-19 cells, providing a promising therapeutic drug for the treatment of diabetic retinopathy (28).

Consistent with the above observations, oxidative stress plays a significant role in the development and progression of diabetes mellitus (DM) and its complications. Mitochondrial dysfunction is often associated with abnormal reactive oxygen species (ROS) production, and elevated ROS levels are linked to insulin resistance (IR) and islet dysfunction in type 2 diabetes mellitus (T2DM).Accumulating evidence suggests that DMY possesses a strong antioxidant effect with relatively good efficacy, indicating that natural antioxidant DMY can be clinically used as an adjunctive or second-line agent for preventing and treating diabetes and its complications.

### 2.5 DMY inhibits the secretion of inflammatory cytokines

Patients with type 2 diabetes mellitus (T2DM) often exhibit a mild chronic inflammatory state, characterized by elevated levels of cytokines and adipokines, as well as activation of pro-inflammatory pathways (58). Concurrently, DMY can directly target peripheral tissues to improve diabetic complications’ symptoms. Numerous reports have demonstrated that the upregulation of pro-inflammatory cytokine expression plays a crucial role in the development of T2DM complications, such as nephropathy, neuropathy, retinopathy, and various cardiovascular diseases. Among these complications, pro-inflammatory cytokines like interleukin-18 (IL-18), interleukin-1β, and tumor necrosis factor α (TNF-α) have been implicated in promoting insulin resistance (IR), impairing β cell function, and inducing cell apoptosis (59). Diabetic neuropathic pain (DNP) and depression are the most common and intractable chronic complications of DM, affecting nearly 25% of diabetic patients (60). Studies have found that DMY could inhibit brain-derived Neurotrophic Factor levels and the expression of Tyrosine Kinase receptor B in the nervous system, and thus improved DNP and depression by reducing the levels of IL-1β and TNF-α in the spinal cord (29). It prevents the inflammation induced tissueinjury by targeting P2X7 receptor (61) and BDNF/TrkB pathway, to reduce levels of pro-inflammatory factors (29). Guan et al. have also found that DMY could directly reduce the expression of P2X7 receptor, down regulate the activation of ERK1/2 pathway, and reduce the release of the inflammatory factor TNF and IL-1β in rats with DNP and depression (30). In addition, one group showed that DMY exerted an anti-inflammatory effect by reducing the levels of IL-6 and TNF-α, and also improved mitochondrial dysfunction through significant enhancement of cellular ATP content, citrate synthase activity and complex I/II/III/IV/V level, thus preventing from experimentally diabetic cardiomyopathy in STZ-induced diabetic mice (14). Another clinical trial found that DMY significantly improved IR in non-alcoholic fatty liver disease (NAFLD), regulated glucose and lipid metabolism, slowed the progression of hepatic steatosis, reduced blood levels of TNF-α, cytokeratin 18 and fibroblast growth factor 21. DMY also improved glucose and lipid metabolism and various biochemical indexes in patients with NAFLD, thus improving blood glucose levels (62).

Liu et al. found that the release of proinflammatory factor HMGB1 in plasma of diabetic rats was increased, and the expression of HMGB1 and phosphorylated NF-κB p65 protein in myocardial tissue was up-regulated, while the expression and release of HMGB1 and phosphorylated NF-κB p65 protein were significantly inhibited in DMY treated diabetic rats. It is reported that DMY may improve cardiomyopathy in diabetic rats by inhibiting the phosphorylation of HMGB1 and NF-κB p65 to reduce inflammatory responses (10). Researchers have shown that DMY reduced HFD-induced inflammatory cytokines (IL-1β, IL-6, TNFα, and MCP1) in the obese mice and could activate the PLC-CaMKK-AMPK signal pathway against inflammation-induced insulin resistance by targeting phospholipase C in peripheral tissue (32).

In recent years, ample research has indicated that the pathogenesis of T2DM is also closely linked to systemic low-grade chronic inflammation. Many inflammatory factors, such as TNF-α, IL-6 and C-reactive protein are not only directly involved in IR, but also closely associated with diabetic macro-vascular complications. The latest study indicated that DMY suppressed the expression of inflammatory factors, indicating that DMY would attenuate inflammatory responses and thus improve diabetes and relevant complications. Previous study have found that liver is one of the target organ of DMY, and it alleviated nonalcoholic fatty liver disease (NAFLD) via regulating lipid/glucose metabolism, probably due to its anti-inflammatory or sirtuins-dependent mechanism (32, 63) DMY was mainly absorbed in gastrointestinal tract, to ameliorate the liver inflammatory activity induced by DM, latest study has found that a long-circling DMY encapsulated liposome with good bioavailability that could target the liver in the long term and sustain DMY delivery. The primary finding of this study was that DMY encapsulated liposomes significantly reduced the liver inflammation induced by exhaustive exercise by promoting macrophage polarization from the M1 to M2 subtype by activating the SIRT3/HF-1α signaling pathway. Hence DMY may provide a novel insight into the research of the anti-diabetic mechanism from anti-inflammatory in clinical practice and lays a foundation for the further development of active components of Chinese herb for treating diabetes.

## 3 Perspective

Diabetes mellitus (DM) comprises a group of chronic metabolic diseases characterized by chronic hyperglycemia resulting from defects in insulin secretion, insulin response, or both. DMY (dihydromyricetin), on the other hand, exerts protective or preventive effects in various systems, including the cardiovascular system, liver, musculoskeletal system, nervous system, and metabolic and endocrine diseases like DM. These beneficial effects are achieved through antioxidant, anti-inflammatory, anti-apoptotic, and other pathways. The underlying mechanism is related to the AMPK/mTOR/PGC-1α, MEK/ERK, etc. signaling pathway. Meanwhile, DMY can also alleviate DM and its complications such as diabetic foot, diabetic nephropathy, diabetic retinopathy and so forth by reducing inflammation, enhancing autophagy, and alleviating ROS via mainly targeting AMPK.

AMPK is an enzyme that plays a crucial role in cellular energy homeostasis, which exists as a heterotrimeric protein consisting of a catalytic α-subunit, regulatory β- subunits and γ-subunits. The α-subunit is the main catalytic subunit of AMPK. Under stress conditions, AMPK was activated by an increased intracellular AMP/ATP ratio. AMP binds to the AMPKγ subunit, causing conformational changes in the protein and promoting phosphorylation of AMPK on threonine residues (Thr172) (64).

In present studies, AMPK, as a key regulator of DMY regulating systemic energy balance, including regulation of glucose and lipid metabolism, attenuating inflammatory responses, and reducing oxidative stress, etc., has become a research hotspot in the field of T2DM (65). In addition to present signaling pathway discussed in the manuscript, another research further demonstrated that DMY could activate AMPK by inhibiting mitochondrial F1F0-ATPase activity, and then induce autophagy to improve skeletal muscle IR and treat diabetes. DMY could activate the AMPK/mTOR signaling pathway, trigger autophagy and act as the antagonism for high glucose-induced oxidative damage of endothelial cells, making it be a hopeful drug for the treatment of T2DM. Similarly, DMY could also mediate the AMPK-PGC-1α-SIRT3 signaling pathway to improve skeletal muscle insulin sensitivity and promote autophagy by activating the AMPK/mTOR signaling pathway, which can alleviate diabetic kidney injury and even retard the progression of diabetic nephropathy. Thus, these studies suggest that DMY is also a natural AMPK activator, and indicate that AMPK may be a clinical target of DMY in the treatment of DM. A recent clinical study showed that DMY can improve glucose and lipid metabolism in patients with T2DM (66). The major challenge for the translational research of DMY is its small molecular weight and poor solubility, which result in its low bioavailability. Currently, organic polymer materials are incorporated to facilitate the biological utilization of DMY, and to develop a more effective drug for clinical treatment of DM. the latest research have found that adipose tissue is the key organ for DMY to function, adipose tissue is multifunctional and plays an important role in whole-body energy homeostasis in DM, Lipid metabolism disorder is one of the pathological processes of DM (67, 68), it is reported that DMY had the capacity to decrease body weight, improve metabolic disorders of glucose and lipids and increase the consumption of energy by inducing WAT browning and offered new insight into the therapeutic action of this small molecule compound in obesity(69). Although lipid metabolism disorder is one of the pathological processes of diabetes, DMY may also had ability to improve metabolic disorders of glucose and lipids in DM.

DMY not only plays an important role in diabetes by targeting its activator AMPK but also exerts significant effects on its targets in other diseases. Sun et al. used the AD model of rats to verify whether DMY can exert the regulatory roles in AD via up-regulation of AMPK/SIRT1 signaling pathway to inhibit inflammatory responses, hippocampal neuronal apoptosis and ameliorate cognitive function, thus providing novel targets for the clinical treatment of AD (P. 70). Moreover, DMY has been shown to increase SIRT3 expression by activating the AMPK-PGC1α/ERRα signaling pathway, and promote the expression of mtDNA-encoded genes, restore the enzymatic activity of MRCs and increase the mtROS scavenging ability through the SIRT3-dependent mechanism, thus improving the mitochondrial respiratory capacity and redox homeostasis of hepatocyte to prevent NAFLD (57). In osteosarcoma cells, Zhao et al. found that DMY executes anti-tumor effects through the induction of apoptosis in osteosarcoma cells and the potential mechanism may be due to the activation of p38MAPK and AMPKα/GSK-3β/Sox2 signaling pathway (71). It has been reported that DMY ameliorated apoptosis and glucometabolic disorders through the regulation of AMPK/GLUT4 signaling pathway in PC12 cells. Specifically, DMY protected PC12 cells against MG-induced apoptosis and glycometabolic disorders, at least in part by restraining the hyperactivation of p-AMPK activity and normalizing the translocation of GLUT4 from the intracellular compartment, resulting in a balance in glucose uptake (72).

No matter whether DMY functions in diabetes by improving glucose and lipid homeostasis in the liver, adipose tissue, and skeletal muscle, or assists in reducing NAFLD through AMPK/PGCl-α/ERR-α or facilitated tumor cell apoptosis through AMPK/GSK3/SOX2 and regulated the AMPK/GLUT4 signaling pathway in the treatment of diabetic encephalopathy, AMPK has been recognized as the most upstream of these cascades, making AMPK is a key target for DMY in the treatment of these diseases. However, how DMY activates AMPK and whether it also mediates other related signaling pathways such as AMPK-NLRP3 to treat diabetes still requires to be elucidated. In the future, molecular docking and molecular dynamics simulations utilized to verify the association between DMY and its potential targets is full of great significance. Furthermore, exploration of the specific binding sites where DMY activates AMPK, such as Thr172, will provide compelling evidence for AMPK to become an attractive potential target for DMY to treat DM. Despite majority of studies focused on the mechanism and potential functions of DMY in treating DM, further study is needed to determine whether DMY combined with first-line diabetes drugs would exhibit better efficacy.

## 4 Conclusion

This review provides a systematic summary and in-depth discussion of DMY in the context of signal transduction pathways that regulate AMPK or mTOR/autophagy, as well as relevant downstream cascades, such as PGC-1α/SIRT3, MEK/ERK, and PI3K/Akt signaling pathways. Indeed, from a pharmacological standpoint, DMY holds promise as a valuable flavonoid compound with potential to prevent the initiation and progression of several diseases. While hypothesized mechanisms of action have been reported, further comprehensive mechanistic and toxicological studies are essential to advance our understanding of DMY’s effects. According to ClinicalTrials.gov, there are only four clinical studies published on DMY aganist human diseases up to date (**Table 2**), and we find that clinical studies of DMY are very limited, as the low bioavailability of DMY is a challenge. Another reason is the "dose-time-toxic-function" of DMY, which is still unclear. Conducting such studies will be crucial in expediting experimental research and preclinical investigations, ultimately paving the way for DMY to potentially be developed into commercial drugs in the near future.

**Table 2:** Clinical trials for Dihydromyricetin.

Status	Study Title	Conditions	Interventions	Locations
Not yet recruiting	Phase I, Dose-Escalation Study of Dihydromyricetin to Treat Alcohol-Associated Liver Disease	Alcohol-Related Disorders	Drug: Dihydromyricetin	University of Southern California
Active, not recruiting	Effect of Dihydromirycetin on Glycemic Control, Insulin Sensitivity and Insulin Secretion in Type 2 Diabetes Mellitus	Type 2 Diabetes Mellitus	Drug: Dihydromyricetin	Instituto de Terapeútica Experimental y Clínica. Centro Universitario de Ciencias de la Salud. Universidad de Guadalajara Guadalajara Jalisco,Mexico
Completed	Stress-induced Sleep Deficits and a Complementary Therapy	Insomnia Due to Anxiety and Fear	Dietary Supplement DMY Dietary Supplement placebo	Furise Group Co Chengdu,Sichuan,China
Recruiting	Efficacy of the Dietary Food Supplement ALCOFILTRUM in Alleviating Alcohol Hangover Symptoms	Alcohol Drinking Alcohol Intoxication	Dietary Supplement: ALCOFILTRUM	Clinical Emergency Hospital, Minsk, Belarus

## Author contributions

ZW was in charge of searching all the relative papers and writing this manuscript. ZC was in charge of drawing the picture. ZFY and ZYY gave their valuable and professional suggestions, guided in organizing and drafting this manuscript and provided funding. ZFY reviewed the manuscript. All authors contributed to the article and approved the submitted version.
